# Lip Pressure, Bite Force and Denture Use as Predictors of Oral Frailty in Physically Active Older Adults: A Cross-Sectional Study

**DOI:** 10.3390/dj14030152

**Published:** 2026-03-09

**Authors:** Catarina Colaço, Inês Caetano-Santos, José Brito, Vanessa Machado, Angel Lobito, José João Mendes, Selma Siessere, Simone Cecílio Hallak Regalo, Luciano Maia Alves Ferreira

**Affiliations:** 1 Egas Moniz Center for Interdisciplinary Research (CiiEM), Egas Moniz School of Health & Science, 2829-511 Almada, Portugal; isantos@egasmoniz.edu.pt (I.C.-S.); jbrito@egasmoniz.edu.pt (J.B.); vmachado@egasmoniz.edu.pt (V.M.); alobito@egasmoniz.edu.pt (A.L.); jmendes@egasmoniz.edu.pt (J.J.M.); lucianomaia@egasmoniz.edu.pt (L.M.A.F.); 2 Neuromodulation and Pain Lab (NEUROPAIN), Egas Moniz School of Health & Science, 2829-511 Almada, Portugal; 3 Faculty of Dentistry of Ribeirão Preto, University of São Paulo (FORP/USP), 14040-904 São Paulo, Brazil; selmas@forp.usp.br (S.S.); simone@forp.usp.br (S.C.H.R.)

**Keywords:** oral frailty, aging, older adults, geriatric assessment

## Abstract

**Background:** Oral frailty is an emerging determinant of late-life disability. While objective functional measures have been proposed as key indicators, their combined role in predicting frailty among physically active older adults remains unclear. Therefore, this study aimed to investigate the association between the presence of oral frailty and lip pressure, bite force, and denture use. **Methods:** This cross-sectional study included 192 participants aged 60 years or older from Brazil (*n* = 131) and Portugal (*n* = 61), all physically active and with ≥20 natural or rehabilitated teeth. Data were collected through a questionnaire on sociodemographic data and the Oral Frailty Index-8. The clinical assessment included lip pressure, bite force, and denture use. Multiple logistic regression identified independent predictors; model fit and discrimination were examined using the Hosmer–Lemeshow test and ROC curve. **Results:** Participants were mainly female (83.3%), mean age ≈72 years; 76% used dentures and frailty prevalence was ≈49%. Higher lip pressure (OR = 0.986, 95% CI = [0.973–0.999]) and higher bite force (OR = 0.925, 95% CI = [0.885–0.967) were independently protective, whereas denture use (OR = 6.898, 95% CI = [2.994–15.895]) markedly increased oral frailty odds. The model showed good discrimination (AUC 0.779). **Conclusions:** Even small increases in lip pressure and bite force reduced the likelihood of frailty, while denture use identified individuals at substantially higher risk. These findings highlight orofacial muscle strength and masticatory capacity as core components of oral frailty and support incorporating lip pressure and bite force testing into multidimensional frailty assessment and targeted rehabilitation.

## 1. Introduction

Global population ageing is increasing, with the number of people aged 60 years or over rising from about 258 million in 1980 to 771 million in 2022 and projected to reach 1.6 billion by 2050, representing roughly one quarter of humanity [1,2,3].

Population ageing has led to a growing prevalence of frailty, a multidimensional geriatric syndrome characterized by diminished physiological reserve and increased vulnerability to stressors that predispose older adults to disability, hospitalization and even mortality [4,5,6]. Frailty is now recognized as a key target for the need for prevention and early intervention in geriatric care as timely identification of at-risk individuals can mitigate functional decline and adverse outcomes. Recent consensus and literature reviews emphasize that frailty is a dynamic construct shaped not only by comorbidities, nutrition and physical activity, but also by cognitive, social and oral health domains [6,7,8,9,10,11].

Within this broader framework, oral frailty has emerged as a conceptual bridge linking deterioration of oral function with systemic frailty and late-life disability [6,8,10]. In 2020, the Japan Dental Association described oral frailty as a mild decline in oral function, accompanied by declines in physical and mental function, occurring in the reversible stage and the early stage of frailty [12]. Recent reviews report that oral frailty is common among community-dwelling older adults and is associated with functional limitations and care dependence, supporting its role as an early warning sign of broader health deterioration [6,8,11,13].

Several epidemiological evidence indicates that poor oral health and impaired oral function are consistently associated with frailty and physical performance decline in later life [11]. Recent literature has identified robust associations between frailty status and indicators such as number of teeth, chewing ability, denture characteristics and clinical oral disease, across different frailty instruments and settings. More recent analyses strengthen the link between compromised oral function and higher odds of physical frailty, reinforcing recommendations to integrate oral assessment into comprehensive geriatric evaluation [8,14,15,16].

Specific oral functional parameters, including occlusal (bite) force, tongue or lip pressure and masticatory performance, have attracted attention as objective and quantifiable markers within the construct of oral frailty [11]. Cross-sectional and longitudinal studies suggest that reduced occlusal force and decreased tongue or lip pressure are associated with prevalent and incident frailty, as well as with related conditions such as sarcopenia, dysphagia and functional dependency [17]. Recent work further proposes bite force as a potential frailty biomarker alongside handgrip strength, reflecting neuromuscular integrity while being less influenced by overall body size and composition [7,14,18,19].

In parallel, research on masticatory performance, denture rehabilitation and nutrition shows that impaired chewing is linked to suboptimal dietary intake, sarcopenia and frailty, and that denture treatment can partly restore function [8,12]. Observational studies indicate that reduced masticatory performance predicts frailty even after adjustment for nutritional variables, suggesting that mastication may influence frailty through both nutritional and non-nutritional pathways such as sensory feedback and social participation. Literature also reported that denture characteristics and perceived chewing difficulty are associated with frailty status, although improvements in denture fit do not invariably translate into better nutritional or functional outcomes [15,17,18,20,21,22]. In addition, relatively few analyses have jointly examined parameters such as bite force, lip or tongue pressure and denture use within the same multivariable model, even though these variables may capture distinct facets of oral frailty and potentially interact [6,8,14,15,16].

This evidence gap limits the integration of oral functional assessment into frailty screening algorithms and clinical decision-making, as clinicians still lack clear data on which oral measures add unique prognostic value beyond established frailty determinants. Without robust multivariable evidence, it remains uncertain whether routine measurement of bite force or lip pressure, or documentation of denture use, should be prioritized in geriatric practice focused on frailty identification and management. It also constrains the development of targeted, interdisciplinary interventions that position oral function as a modifiable lever within comprehensive frailty prevention and rehabilitation strategies [6,8,14,15,23].

Therefore, this study seeks to investigate the association between the presence of oral frailty and qualitative factors, such as bite force, lip pressure, and the denture use, in physically active older adults living in Brazil and Portugal.

## 2. Materials and Methods

### 2.1. Study Design and Participants

This observational cross-sectional and multicenter study included a convenience sample of 192 patients (*n* = 192), with 131 Brazilians (*n* = 131) from CASI project (Center for Support and Health of the Elderly, Batatais/SP) and 61 Portuguese (*n* = 61) from the project “Sempre a Mexer” (Sesimbra, Setúbal), between April and September 2024. Inclusion criteria included age of 60 years or older, being able to read, write, speak and understand Portuguese, being able to comply with the study protocol, not having disabilities such as blindness, deafness or dementia, with at least 20 natural or functionally rehabilitated teeth (with fixed or removable denture) and perform 150 or more minutes of physical activity per week, according to the International Physical Activity Questionnaire (IPAQ) [24,25]. All individuals who did not meet the inclusion criteria and those who refused to participate and sign informed consent were excluded. The reporting of data followed the STROBE checklist [26].

### 2.2. Ethical Aspects

The project was approved by the Research Ethics Committee of Egas Moniz School of Health & Science (No. 6.787.851, CAAE: 79222024.8.0000.5419, on 30 June 2022) and conducted according to the tenets of the Declaration of Helsinki, as revised in 2013. All participants were informed about the objectives of the study and signed an Informed Consent Form. Participation was voluntary, with a guarantee of confidentiality and freedom to withdraw at any time, without losses or costs. The data were collected anonymously by coding and were only intended for statistical processing and/or publication, maintaining the anonymity and confidentiality of the participants.

### 2.3. Data Collection

Data were collected through a questionnaire and a clinical assessment always by the same trained examiner (L.M.A.F.). The self-reported questionnaire collected information on sociodemographic information (age, biological sex and country) and oral frailty. The clinical assessment included lip pressure, bite force, body Mass Index (BMI) and the use of removable or fixed partial denture.

#### 2.3.1. Oral Frailty (Dependent Variable)

Oral frailty was measured based on the OFI-8, an eight-item screening tool that evaluates oral health-related behaviors and frailty constructs, developed through expert consultation. The OFI-8 assigns double weighting to the three highest-priority items representing the core components of oral frailty: “tooth loss,” “subjective chewing difficulties,” and “subjective swallowing difficulties”. The OFI-8 total score ranges from 0 to 11 points, where higher values denote oral frailty [27]. In this study, the translated and validated Portuguese version of the OFI-8 was used (OFI-8/PT) [28].

#### 2.3.2. Sociodemographic Data

Sociodemographic data included biological country (Brazil or Portugal), sex (male/female) and age (years).

#### 2.3.3. Lip Pressure Measurement

The PLL Pró-Fono is a portable instrument designed to measure lip and tongue pressure using an air-filled bulb connected to a pressure sensor by a flexible plastic tube. Changes in bulb air pressure are detected by the sensor and converted into pressure values expressed in kilopascals (kPa). To evaluate lip pressure, the methodology was used according to a study by Ramos et al. (2023) [29], where the bulb was placed between the lips so that the labial muscles compressed it during the task. Each participant was instructed to maintain the maximal pressure for 3 s in three separate attempts, with 30 s rest intervals, controlled with a digital stopwatch. After the procedure, the device software automatically provided a report including the individual pressure values for each attempt and the mean value calculated across the three trials.

#### 2.3.4. Bite Force Measurement

Bite force was measured using a mandibular force electric dynamometer from EMG System do Brasil^®^ (São José dos Campos, São Paulo, Brazil), which consists of a bite force transducer (bite plate) connected to a computer that acquires signals in kilograms of force (Kgf). The dynamometer was enclosed in a disposable barrier, and its mechanical components were decontaminated and calibrated in strict accordance with the manufacturer’s specifications. Participants received standardized instructions on how to perform the test, which was first demonstrated by the examiner. Each participant was then allowed for a single familiarization trial with the device before the actual testing procedure commenced. The procedure was performed three times with a 60 s interval between each measurement to allow the muscles to rest. Participants remained seated with their back gently leaning against the back of the chair, their feet flat on the floor, their arms resting on their legs, and their head in a natural position. Upon request, they bit down on the plate with maximum force for 10 s. Participants were instructed to stop immediately if they experienced any unusual pain or discomfort during the test. The evaluator provided verbal cues to ensure the participant maintained the same intensity throughout the evaluation. Signal analysis was performed using software from the same manufacturer based on the period of greatest bite stability. This verified the average graph value in Kgf. The data analysis considered the average of three bite records [7].

#### 2.3.5. Body Composition Measurement

Body composition was assessed by bioelectrical impedance analysis (BIA) with the ACCUNIQ BC310 model (Daejeon, Republic of Korea). Participant identification, age, and sex were entered into the device software, height was obtained using a stadiometer, and weight was measured by the scale, which subsequently generated an automatic report containing the body mass index (BMI), expressed in kilograms per square meter (kg/m^2^). Participants were evaluated in a fasting state and were instructed to refrain from physical exercise and from consuming beverages with stimulant effects (such as coffee or energy drinks) during the 24 h preceding the measurement. Participants were required to remove all metallic accessories (earrings, rings, belts) and to remain in minimal clothing, barefoot and without socks. Each participant stood on the scale with feet placed parallel over the plantar electrodes, while the arms rested alongside the trunk holding the hand electrodes. The measurement was initiated by pressing the start button simultaneously with both thumbs for approximately 10s, according to the factory settings [30]. For the purposes of this study, BIA was used solely to estimate BMI, while the additional parameters generated by the device will be reserved for analysis in future investigations.

#### 2.3.6. Use of Removable or Fixed Partial Denture

Denture use was recorded if the participant could show or wear removal or fixed denture (“yes”) or not (“no”).

### 2.4. Statistical Analysis

The statistical analysis program IBM SPSS Statistics version 30.0 (IBM, Armonk, NY, USA) was used to analyze all data obtained in this research, using descriptive and inferential statistical analysis methodologies. For descriptive analysis, categorical data were presented as frequency and percentage distributions, and numerical data as mean and standard deviation (SD).

A multiple logistic model has been fit to the data to predict the probability of oral frailty, as defined by the dichotomous variable OFI-8/PT (fragile or nonfragile), using a set of predictors including Country (0 = Brazil; 1 = Portugal), Sex (0 = Female; 1 = Male), Age (years), Lip Pressure (KPa), Bite Force (Kgf), and the Denture use (1 = yes; 0 = no). The overall significance of the model was tested using the “−2 log Likelihood function”, whereas the significance of the predictors included in the model was tested using the Wald statistics. Odds Ratio (OR) and 95% confidence intervals (95% CI) were calculated. A significance level of 5% (*p* ≤ 0.05) was established in all inferential analyses.

Following recommendations in the literature [31], each variable was screened one at the time in order to see which ones are associated with oral frailty, using independence tests for nominal predictors (country, sex and denture use) and t-tests for continuous predictors, using a large significance level (*p* = 0.15). Only variables for which *p* values did not exceed 0.15 were included for further analysis, as was the case of predictors Lip Pressure, Bite Force and Denture use.

The Hosmer–Lemeshow test was used to assess model calibration, while the Receiver Operating Characteristic (ROC) curve and Area Under the Curve (AUC) were used to evaluate model discrimination.

## 3. Results

Descriptive data of the study participants is presented in Table 1. The sample included 192 participants (*n* = 192), with the mean age of 72.3 (SD = 6.9) years; range 55–92, mostly female (83.3%) and predominantly from Brazil (68.2%). The mean BMI was 28.2 kg/m^2^ (SD = 5.2). Functional measurements showed substantial variability. Mean lip pressure was 58.7 kPa (SD = 22.9), and bite force was 18.8 Kgf (SD = 5.0). Most participants (76%) used denture. Oral frailty was present in 48.9% of the sample.

Descriptive data for study participants according to the country are presented in Table 2. The sample included 131 participants from Brazil, with a mean age of 72.3 years (SD = 6.6). Most of the participants were women (82.4%) and the mean BMI was 28.6 kg/m^2^ (SD = 5.4). Functional measurements showed that the mean lip pressure was 56.3 kPa (SD = 26.7) and the mean bite force was 17.8 Kgf (SD = 7.7). Most participants (68.7%) wore denture, and oral frailty was present in 49.6% of the sample. The Portuguese sample included 61 participants (*n* = 61), with a mean age of 72.1 years (SD = 7.5). Most participants were women (85.2%), and the mean BMI was 27.4 kg/m^2^ (SD = 4.5). Functional measurements showed that the mean lip pressure was 60.7 kPa (SD = 27.5), and the mean bite force was 18.4 Kgf (SD = 8.4). Most participants (80.3%) wore dentures, and oral frailty was present in 47.5% of the sample.

Country, sex, and age were not significantly associated with oral frailty in the univariate screening analysis (*p* > 0.15) and were therefore not included in the final multivariable logistic regression model. When the full model was fitted to the data, its overall significance was observed (*p* < 0.001), while Lip Pressure (*p* = 0.029), Bite Force (*p* = 0.002) and the Denture use (*p* < 0.001) were significant predictors of oral frailty. Table 3 describes the logistic model obtained from the data.

From Table 3, it can be concluded that the chances of oral frailty decrease by 1.4% with each 1 Kpa increase in Lip Pressure, whereas those chances decrease by 6.5% with each 1 Kgf increase in Bite Force. In addition, the chances of oral frailty of subjects using dentures are approximately 5.4 times those of subjects who do not use denture.

Analysis of plots of squared studentized residuals versus predicted probabilities has shown the existence of two residuals between 2 and 3, suggesting moderate influence from those observations. The removal of those cases did not change the significance of the overall model, nor the significance of predictors. Of the predictors, only denture use had an effect on the dependent variable, with the magnitude of the effect on the likelihood of frailty changing from 5.4 to 6.9.

Since there was also an increase in the significance of the Hosmer–Lemeshow test (from 0.080 to 0.352) suggesting model fit improvement with the removal of those observations, we chose to interpret the final model under those conditions, although the overall conclusions on the effects of predictors are the same. Table 4 shows the model structure after removal of the two observations.

**Figure 1:**
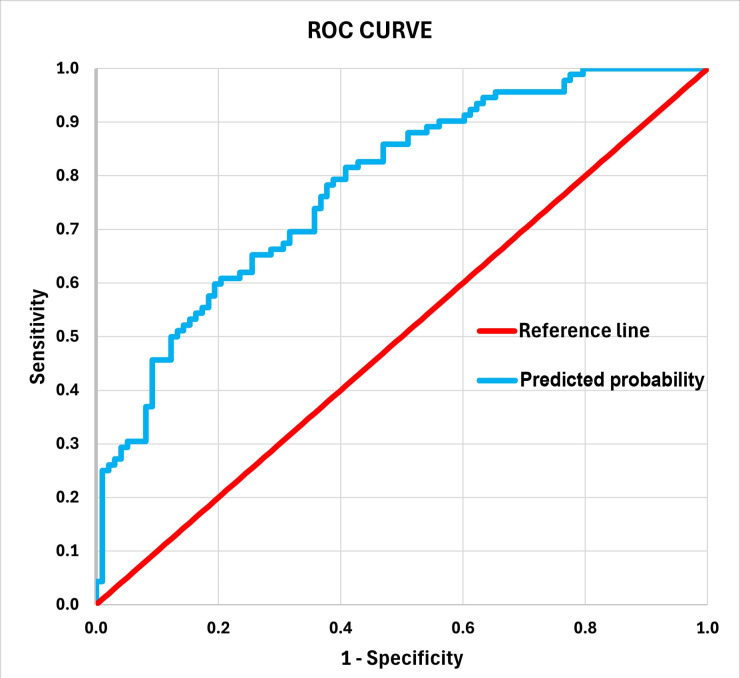
ROC Curve for the predictive model of oral frailty.

For this model, a Receiver Operating Characteristic curve (ROC curve) was plotted (Figure 1) based on a range of sensitivities and specificities over possible cut-off values in predicted probabilities of frailty and the area under the curve (AUC) was estimated to be AUC = 0.779 (*p* < 0.001, against the null hypothesis that the true area is 0.5).

## 4. Discussion

To the best of our knowledge, few studies have specifically examined lip pressure, bite force decline, and denture use in relation to oral frailty among physically active older adults, and available evidence remains limited in this population. The results of this study indicate a high prevalence of oral frailty, with around half of the participants in both countries being classified as frail by the OFI-8/PT, suggesting a substantial impact of this condition on community-dwelling older adults.

Our results revealed that the likelihood of being frail decreases with both increased lip pressure and bite force. Conversely, denture use was found to be associated with a roughly increase in the probability of frailty. These findings reinforce the idea that oral function plays a central role in determining frailty, which is in line with reviews describing oral frailty as the result of the interaction between masticatory capacity, orofacial muscle strength and dental condition throughout life [8,32,33].

The inverse association between lip pressure and frailty is particularly relevant in the context of oral frailty, a concept which includes the hypofunction of perioral and lingual muscles as an early component of functional decline [32]. Reduced lip pressure compromises lip sealing, favoring the leakage of food and liquids from the front of the mouth, increasing the time taken for food to be transported in the mouth, and consequently leading to compensatory strategies such as choosing softer and less varied textures. These dietary adjustments tend to reduce protein and fiber intake, contributing to sarcopenia, malnutrition, and worsening systemic frailty [34,35]. Studies have demonstrated that interventions involving orofacial exercises can lead to notable improvements in lip and tongue strength, as well as enhancements in swallowing parameters. In certain studies, these interventions have also been shown to reduce indicators of oral frailty. [36,37]. This suggests that the orofacial musculature is a promising therapeutic target.

Similarly, the relationship between greater bite force and a lower probability of frailty supports the evidence linking masticatory function to overall health. Bite force is an indicator of overall masticatory capacity. It depends on the number and distribution of teeth, muscle integrity, and neuromuscular coordination [7]. Reduced bite force has been associated with difficulty chewing hard foods, limited food choices, and poorer nutritional status. Longitudinal studies show that older adults with lower bite force are at greater risk of developing physical frailty and functional decline, even when controlling for age and comorbidities [38]. This reinforces the idea that effective chewing is an important reserve for healthy aging.

The most consequential outcome of the model, nevertheless, is the effect of denture use: the risk of frailty is approximately 5.4 times higher among users than non-users. This finding may seem paradoxical since denture rehabilitation is designed to restore function. However, several studies report that removable denture use, especially conventional complete dentures, is often associated with poor chewing ability, limited food intake, and a lower quality of life, particularly when the dentures are unstable or poorly fitted. Additionally, denture use is an indirect marker of edentulism, or severe tooth loss. This reflects the accumulation of oral disease throughout life and often indicates reduced access to care [39,40]. Population studies have shown that edentulous elderly individuals and denture wearers exhibit greater overall frailty, higher mortality rates, and poorer self-perceived health, suggesting that dentures, particularly when dysfunctional, may indicate a state of accumulated vulnerability rather than serving as a rehabilitative solution [41,42].

In the present study, sociodemographic status was not found to be a major risk indicator for oral frailty, which may be related to the fact that all participants were of a similar age. The participants were on average around 70 years old, showing a homogeneous distribution and a moderate standard deviation (SD), which is consistent with advanced age and community dwelling. The literature confirms that frailty and oral frailty prevalence rise sharply after the age of 70, which explains the high rates observed [12,13,43]. However, age was non-significant in the final regression model, indicating that within this age range, oral function, muscle strength, and denture status exert greater influence.

The sample shows a clear female majority in both Brazil and Portugal, reflecting global trends of greater female longevity, chronic disease survival, and higher participation in health screenings and research assessments [7,12,44,45,46]. This overrepresentation may overestimate overall frailty prevalence, as women are generally more vulnerable [7,12,44,46].

The country variable distinguished participants from Brazil and Portugal. Comparative studies show that oral health indicators such as tooth loss and denture use mainly reflect biological aging and accumulated socioeconomic conditions, with health system differences becoming less relevant once advanced tooth loss thresholds are reached. Therefore, the limited contribution of the country to our model suggests that oral frailty risk in this age group is primarily driven by effective oral function, rather than broader contextual factors [8,47].

### Limitations

This study has some limitations that should be acknowledged. In interpreting these findings, it is important to recognize that the cross-sectional design precludes robust inference about temporal or causal relationships between the variables examined. Future research employing longitudinal cohorts or intervention trials that follow participants over time could deepen understanding of this phenomenon and allow the inclusion of additional explanatory factors. Particularly regarding sociodemographic and the characterization of oral variables, the sample is predominantly female, consistent with women’s longer life expectancy and greater propensity to participate in health studies. However, this may bias estimates of frailty prevalence. Ideally, future studies would recruit gender-balanced samples and perform stratified analyses. The sample size adopted was a convenience sample for the period of the study, so representativeness may be limited. Whenever sample size allows, these analyses should consider interactions between sex and oral variables. Some studies suggest that the relationship between tooth loss, masticatory capacity, food intake, and nutritional status may differ between men and women.

Another limitation relates to how the variable use of denture was defined. In this study, the variable was only treated in a dichotomous manner (yes/no), and no distinction was made based on the type of denture (e.g., partial or total; conventional or implant-supported), degree of stability and retention, or level of user satisfaction. These parameters are identified in the literature as determinants of masticatory function and the impact of rehabilitation on quality of life. This methodological choice stems from the main objective of exploring oral frailty using the OFI-8/PT. This instrument already includes an item on denture to use and serve as the basis for selecting the variables of interest. This choice favors comparability with other studies based on the same index. However, this simplification limits the ability to distinguish between functional and nonfunctional denture, as well as the ability to isolate the effect of underlying tooth loss. Therefore, the strong association found between denture use and frailty should be interpreted with caution and investigated further in future studies with a more detailed denture characterization.

Despite the limitations, the study contributes to the emerging evidence that specific oral functional parameters and denture status are key components of the oral frailty construct and that they are closely associated with the probability of being classified as frail in later life. The consistent results between Brazil and Portugal indicate that these relationships may be robust across different cultural and health-care contexts, reinforcing the universal relevance of oral function for healthy ageing. Future research should aim to validate similar predictive models in larger, more diverse cohorts, to test the sensitivity and specificity of different cut-off points for lip pressure and bite force, and to explore the incremental value of oral frailty assessment when added to existing geriatric screening tools. Interventional trials are particularly needed to examine whether strategies such as intensive prosthodontic rehabilitation, implant-supported denture, structured orofacial exercise programs and comprehensive dietary counselling can effectively improve oral function, reverse or attenuate oral frailty and ultimately translate into better general health outcomes for older adults.

## 5. Conclusions

This study identified a high prevalence of oral frailty among community-dwelling older adults in Brazil and Portugal, with bite force, lip pressure, and denture use emerging as the strongest predictors. Small increases in lip pressure and bite force substantially reduced frailty likelihood, while denture use identified individuals at significantly higher risk. These findings highlight orofacial muscle strength and masticatory capacity as core components of oral frailty, supporting the integration of lip pressure and bite force testing into multidimensional frailty assessments and targeted rehabilitation strategies to preserve functional oral health in aging populations.

## Figures and Tables

**Table 1:** Descriptive data on sociodemographic, oral frailty, lip pressure, bite force, BMI and denture use.

Variable		*n*	%	Mean ± SD
Country	Brazil	131	68.2%	
	Portugal	61	31.8%	
Sex	F	160	16.7%	
	M	32	83.3%	
Age (years)				72.3 ± 6.9
Oral Frailty	Fragile	94	48.9%	
	Nonfragile	98	51.1%	
Lip pressure (KPa)				58.7 ± 22.9
Bite force (Kgf)				18.8 ± 5.0
BMI (kg/m^2^)				28.2 ± 5.2
Denture use	Yes	139	76%	
	No	52	24%	

**Table 2:** Descriptive data according to country.

Country	Variable		*n*	%	Mean ± SD
Brazil	Sex	F	108	82.4%	
		M	23	17.6%	
	Age (years)				72.3 ± 6.6
	OFI-8/PT	Fragile	65	49.6%	
		Nonfragile	66	50.4%	
	Lip pressure (KPa)				56.3 ± 26.7
	Bite force (Kgf)				17.8 ± 7.7
	BMI (kg/m^2^)				28.6 ± 5.4
	Denture use	Yes	90	68.7%	
		No	41	31.3%	
Portugal	Sex	F	52	85.2%	
		M	9	14.8%	
	Age (years)				72.1 ± 7.5
	OFI-8/PT	Fragile	29	47.5%	
		Nonfragile	32	52.5%	
	Lip pressure (KPa)				60.7 ± 27.5
	Bite force (Kgf)				18.4 ± 8.4
	BMI (kg/m^2^)				27.4 ± 4.5
	Denture use	Yes	49	80.3%	
		No	12	19.7%	

**Table 3:** Logistic model obtained from the data.

	B	S.E.	Wald	df	Sig.	Exp(B)	95% CI for Exp(B)	
							Lower	Upper
Lip Pressure (kPa)	−0.014	0.006	4.792	1	0.029	0.986	0.974	0.999
Bite Force (Kgf)	−0.068	0.022	9.841	1	0.002	0.935	0.896	0.975
Use of Denture	1.685	0.397	18.016	1	<0.001	5.392	2.477	11.739
Constant	0.718	0.599	1.435	1	0.231	2.050		

**Table 4:** Variables in the Equation after removal of the two observations.

	B	S.E.	Wald	df	Sig.	Exp(B)	95% CI for Exp(B)	
							Lower	Upper
Lip Pressure (kPa)	−0.014	0.007	4.648	1	0.031	0.986	0.973	0.999
Bite Force (Kgf)	−0.078	0.022	12.067	1	<0.001	0.925	0.885	0.967
Use of Denture	1.931	0.426	20.561	1	<0.001	6.898	2.994	15.895
Constant	0.664	0.618	1.154	1	0.283	1.943		

## Data Availability

The data presented in this study are available on request from the corresponding author due to privacy and ethical reasons.
